# High-resolution genomics uncovers region-specific evolution and virulence of ocular *Streptococcus pneumoniae*

**DOI:** 10.1128/spectrum.03291-25

**Published:** 2026-02-03

**Authors:** Xi Xiang, Mengke Ye, Shouyuan Chen, Lin Liu, Tingting Hong, Dongping Hu, Lihong Ge, Ying Fu, Feng Zhao, Yanfei Wang, Lihong Bo, Jun Lu, Yan Jiang, Yunsong Yu, Xueqing Wu

**Affiliations:** 1 Department of Clinical Laboratory, Affiliated Jinhua Hospital, Zhejiang University School of Medicine, Jinhua, Zhejiang, China; 2 Department of Infectious Disease, Sir Run Run Shaw Hospital, Zhejiang University School of Medicine 26441, Hangzhou, China; 3 Key Laboratory of Microbial Technology and Bioinformatics of Zhejiang Province, Hangzhou, Zhejiang, China; 4 Regional Medical Center for National Institute of Respiratory Diseases, Sir Run Run Shaw Hospital, Zhejiang University School of Medicine 26441, Hangzhou, China; 5 Laboratory Medicine Center, Department of Clinical Laboratory, Zhejiang Provincial People's Hospital, Affiliated People's Hospital, Hangzhou Medical College 117839, Hangzhou, Zhejiang, China; 6 Department of Infectious Disease, Affiliated Dongyang Hospital of Wenzhou Medical University 117858 , Dongyang, Zhejiang, China; 7 Department of Clinical Laboratory, Children’s Hospital, Zhejiang University School of Medicine, National Clinical Research Center for Child Health, Hangzhou, Zhejiang, China; 8 Department of Clinical Laboratory, Sir Run Run Shaw Hospital, School of Medicine, Zhejiang University, Hangzhou, Zhejiang, China; 9 Key Laboratory of Precision Medicine in Diagnosis and Monitoring Research of Zhejiang Province, Hangzhou, Zhejiang, China; 10 Department of Clinical Laboratory, Quzhou People’s Hospital, The Quzhou Affiliated Hospital of Wenzhou Medical University 91619, Quzhou, China; Meijo Univ, Nagoya, Japan

**Keywords:** *Streptococcus pneumoniae*, ocular infection, virulence factors, recombination, evolution

## Abstract

*Streptococcus pneumoniae* is the leading cause of sight-threatening ocular infection. However, comprehensive molecular epidemiological analyses that focus on ocular pneumococci are limited. We performed a high-resolution whole-genome analysis (WGS) of ocular pneumococcal isolates from the Zhejiang province of China and a global data set (*n* = 58). Using latex agglutination and the Quellung reaction, nine serotypes were identified, with PCV13 covering 54.5% of the Chinese isolates, and no non-typeable (NT) strains were found in conjunctivitis. Illumina short-read data were assembled *de novo* and submitted for resistance and virulence gene BLAST, phylogenetic analysis, recombination prediction, and single-nucleotide polymorphism (SNP) analysis. Phylogenetically, the Chinese isolates showed a sporadic distribution. All Chinese strains carried the *ermB* gene for macrolide resistance, rather than *mefA,* in strains from Western countries. Virulence gene analysis revealed that no *zmpC* gene was identified in Chinese pneumococcal strains associated with keratitis and conjunctivitis. A marked accumulation of SNPs and mosaic blocks in Asian isolates is strongly associated with the acquisition of region-specific alleles encoding the ATP-dependent protease *SP_0338* and the UV-damage repair enzyme *uvrB*. Interestingly, the DNA integrity gene *SP_1247* has emerged as a globally conserved and functionally essential determinant of ocular *S. pneumoniae*. These findings underscore that genes involved in DNA integrity surveillance and UV stress response are enriched for non-synonymous mutations and might be a virulence prerequisite for pneumococci colonizing the UV-exposed ocular surface. Such features should guide the development of vaccines, antibiotics, and diagnostics for pneumococcal eye infections.

**IMPORTANCE:**

Due to the lack of studies focusing on ocular pneumococcus other than in the United States, little is known about the molecular epidemiology of *S. pneumoniae*, which causes ocular infections in other regions worldwide. This study provides the first high-resolution genomic map of ocular *S. pneumoniae*, revealing Asian-specific variability in UV-damage repair (uvrB) and global DNA integrity (SP_1247) genes that may be essential for survival on UV-exposed ocular surfaces. By linking ermB-driven macrolide resistance, the absence of zmpC, and unique serotype and recombination distribution to these UV-adaptation signatures, this work redefines ocular pneumococci as a distinct ecotype whose regional evolution must be incorporated into future vaccines, antibiotic guidelines, and rapid molecular diagnostics to prevent blindness-causing eye infections.

## INTRODUCTION

*Streptococcus pneumoniae* is an opportunistic, pathogenic, Gram-positive bacterium that causes various infections. These include invasive pneumococcal diseases (IPD), such as bacterial pneumonia, sepsis, and meningitis, and non-invasive pneumococcal diseases (NIPD), including upper respiratory tract infections, otitis media, and ocular infections (1, 2). The unique immunological characteristics of the ocular environment result in distinct clinical features and epidemiological patterns compared with other pneumococcal infections (3, 4).

Compared with other pneumococcal infections, studies on *S. pneumoniae* that cause ocular infections are sparse and limited to strain identification and clinical settings (5–7). Genome-based molecular epidemiological studies of ocular *S. pneumoniae* have only been conducted in the United States (4, 8, 9), showing that non-encapsulated (NT) *S. pneumoniae* accounts for more than 60% of eye infections. Most conjunctivitis strains are NT strains with pneumococcal virulence factors that differ from others (4). The virulence factors of *S. pneumoniae* include capsules, toxins, and surface proteins (10, 11). In keratitis, pneumolysin, neuraminidase, and zinc metalloproteinase C are necessary for pneumococcal pathogenesis (12–14). Polysaccharide capsules and pneumolysins are important in pneumococcal endophthalmitis (15, 16). However, comprehensive genetic and virulence research associated with *S. pneumoniae* ocular infections remains unreported in nations and territories other than the United States. It is important to broaden our understanding of the molecular epidemiology and pathogenesis of ocular pneumococcal infections.

Therefore, the current study aimed to investigate drug resistance, serotype distribution, virulence, and molecular epidemiology of ocular pneumococcal strains in three tertiary hospitals in Zhejiang Province, China. To elucidate the evolutionary relationship between *S. pneumoniae* strains causing ocular infections in China in a global context, we conducted whole-genome analysis of ocular pneumococci worldwide.

## MATERIALS AND METHODS

### *Streptococcus pneumoniae* isolation and identification

Eleven *S. pneumoniae* isolates were collected from three tertiary hospitals in Zhejiang, China, from 2010 to 2023. The specimen types included eight eye swabs and three secretions, which are listed in Table 1 for each isolate. All bacterial isolates were initially obtained by culturing a clinical sample on Columbia agar plate at 37°C and 5% CO_2_. Suspected pneumococcal isolates were selected for identification by optochin susceptibility, bile solubility, and *lytA* PCR tests.

**TABLE 1:** Clinical characteristics of patients with *S. pneumoniae* ocular infections and pneumococcal strain profile^*a*^

Case no.	Clinical presentation	Course (day)	Outcomes	Isolate source	Isolate ID	Serotype	MIC (µg/mL)				
							PEN	CRO	ERY	TET	LVX
1	Conjunctivitis	N/A	N/A	Secretion	hz10027	18C	≤0.125	≤0.125	>128	32	1
2	Keratitis	N/A	N/A	Secretion	qz10017	35C	≤0.125	≤0.125	>128	≤0.5	1
3	Keratitis	5	Cure	Eye swab	hz16005	NT	1	1	>128	32	1
4	Keratitis	11	Cure	Eye swab	hz16007	6C	≤0.125	≤0.125	>128	32	1
5	Keratitis	20	Cure	Eye swab	hz16008	17A	≤0.125	≤0.125	>128	32	1
6	Keratitis	38	Cure	Eye swab	hz17006	17F	≤0.125	≤0.125	64	8	1
7	Endophthalmitis	90	Cure	Eye swab	hz17008	6B	≤0.125	≤0.125	>128	32	1
8	Keratitis	6	Cure	Eye swab	hz17016	23F	2	1	>128	16	1
9	Keratitis	4	Cure	Eye swab	hz19005	23F	≤0.125	≤0.125	>128	32	1
10	Dacryocystitis	N/A	N/A	Secretion	qz22021	19F	8	4	>128	64	1
11	Conjunctivitis	8	Cure	Eye swab	jh23073	6B	2	1	>128	32	1

^*a*^ N/A, unavailable; PEN, penicillin; CRO, ceftriaxone; ERY, erythromycin; TET, tetracycline; LVX, levofloxacin.

### Clinical data collection

Clinical data including sex, age, and primary diagnosis of *S. pneumoniae* ocular infections were retrospectively collected from the medical records of three tertiary hospitals (Sir Run Run Shaw Hospital, Quzhou People’s Hospital, and Affiliated Jinhua Hospital of Zhejiang University) in Zhejiang Province, China (Table 1).

### *Streptococcus pneumoniae* serotyping

All identified pneumococcal isolates were serotyped by latex agglutination tests and Quellung reactions (SSI Diagnostica, Denmark) according to the manufacturer’s protocol by three different persons. To avoid subjective errors in microscopic observations, we conducted *in silico* serotyping via whole-genome sequence analysis using the SeroBA software (17).

### Antimicrobial susceptibility test

The minimal inhibitory concentrations (MICs) of penicillin (PEN), ceftriaxone (CRO), erythromycin (ERY), tetracycline (TET), and levofloxacin (LVX) against the isolated pneumococcal strains were tested using the broth microdilution method according to the Clinical and Laboratory Standard Institute (CLSI) protocol, as described previously (18). An *S. pneumoniae* reference strain (ATCC 49619) was used as quality control for each antimicrobial susceptibility test. The MIC of each tested drug was interpreted according to the 2023 Clinical and CLSI Guideline M100-Ed33 (19).

### Whole-genome sequencing and analysis

Before whole-genome sequencing, the QIAamp DNA Mini Kit (Qiagen, Valencia, CA, USA) was used for genomic DNA extraction according to the manufacturer’s protocol. Next-generation sequencing (NGS) was performed using the Illumina HiSeq X 10 platform (Illumina, San Diego, CA). All sequence data were trimmed and quality-controlled using Fastp (20). To confirm the serotyping results from Quellung reactions, we conducted *in silico* serotyping using SeroBA (v0.1.2) (17). Illumina reads were assembled by end-pairing using Shovill (v0.9.0) (21), with a minimum splicing length of 200 bp and a minimum coverage of 10-fold. The final assemblies had N50 values not less than 60 K, and the minimum sequencing depth was 300×. Subsequently, we determined each strain’s sequence type (ST) using a genome against the PubMLST database (https://pubmlst.org) via MLST (22). After a comprehensive search of the PubMLST Pneumococcal Genome Library database, we successfully acquired genome data of 47 pneumococcal strains. These strains were obtained from samples related to ocular infections by filtering the isolation source field using the keyword eye swab. Virulence and antimicrobial resistance genes for all analyzed strains (*n* = 58) were screened using ABRicate (v1.0.0, https://github.com/tseemann/abricate) against VFDB (2020) (23) and ResFinder (2020) (24) databases, respectively. Amino acid substitutions in PEN-binding proteins (PBP1a, PBP2b, and PBP2x) were analyzed using BLAST+ (v2.13.0) (https://github.com/ncbi/blast_plus_docs) against the CDC database (USA) (25). Phylogenetic trees were constructed, and GPSC were assigned using PopPUNK (v2.4.0) (26) and visualized using iTOL (v6) (27). The contigs of each strain were mapped to a reference strain, TIGR4 (AE005672.3), to generate single-nucleotide polymorphism (SNP) calls using Snippy (v4.4.5, https://github.com/tseemann/snippy). Full-core alignment files were generated using Gubbins (v2.4.1) (28) to identify recombination events. SnpEff (v5.2) (29) was used for the SNP analysis of all genes according to the Snippy-generated core.vcf files. The SNP-based phylogenetic tree and recombination events were visualized using RStudio (v2023.12.1+402) (30) and the RCandy package (v1.0.0) (31).

### Statistical analysis

The differences between these four regions (China, North America, Europe, and other Asian countries) in the total SNP number for each isolate, number of SNPs inside recombination, recombination blocks, and ratio of SNPs caused by recombination and mutation (r/m) were tested by one-way ANOVA and Tukey’s multiple comparisons test for post-hoc analysis using GraphPad Prism v9.5.0, where *P <* 0.001 was considered statistically significant.

## RESULTS

### Clinical characteristics of ocular infections caused by *S. pneumoniae*

The demographic and clinical data of the patients diagnosed with ocular infections due to *S. pneumoniae* are summarized in Table 1. We collected 11 cases of pneumococcal ocular infection, three of which were female, and only one case was from a child under five. The data indicated a range of clinical presentations, predominantly keratitis, with two cases of conjunctivitis, one of endophthalmitis, and one of dacryocystitis. The outcomes for most patients were favorable, with most achieving cure, whereas endophthalmitis had the longest treatment duration (90 days).

### *S. pneumoniae* serotype distribution and antimicrobial susceptibility

**Fig 1:**
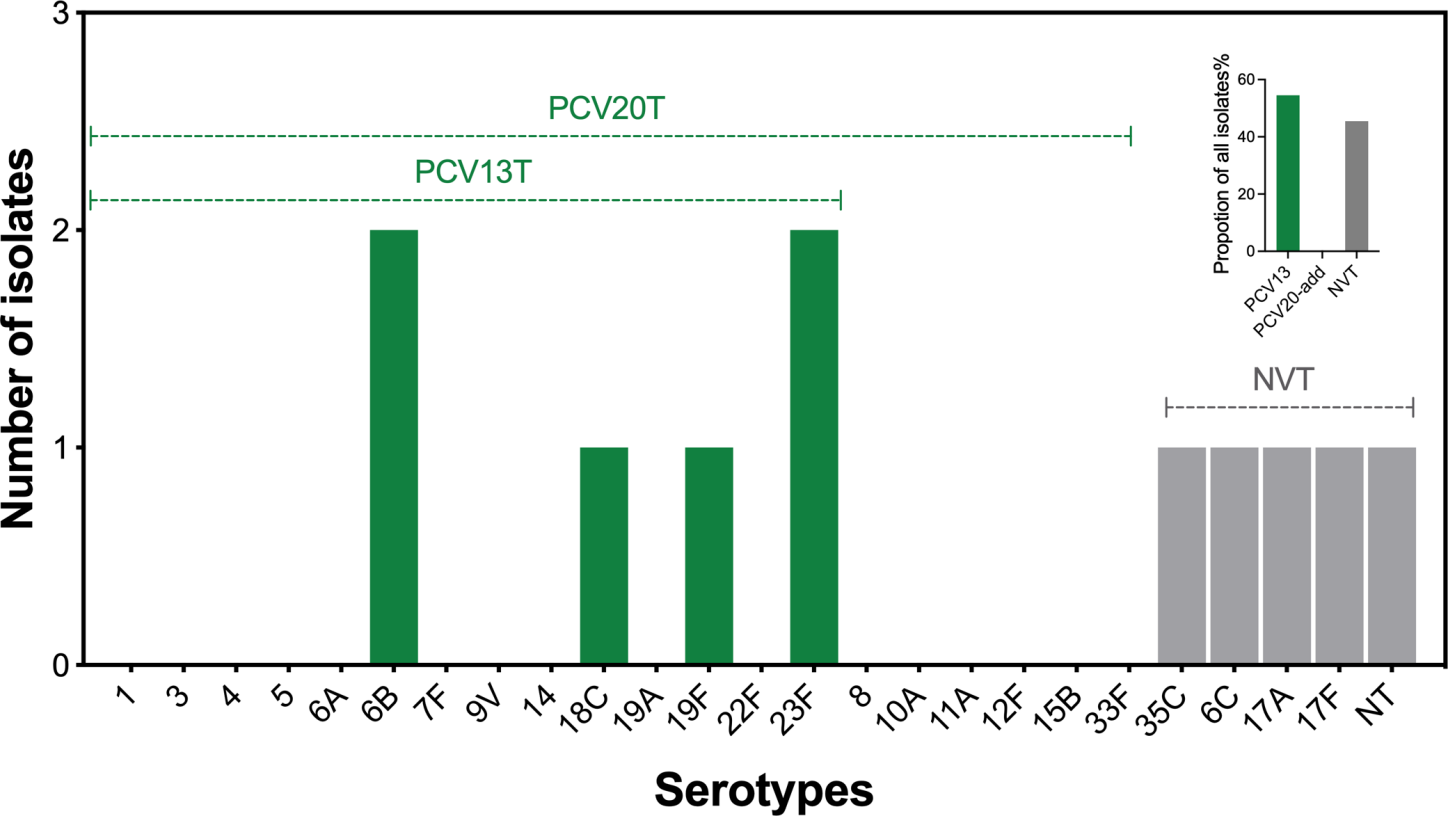
Serotype distribution of ocular *S. pneumoniae* in Zhejiang, China. The serotypes of *S. pneumoniae* strains collected in the current study are presented in the bar figure. The green bars represent the pneumococcal conjugate vaccine types (PCV12T and PCV20T), and the gray bars represent the non-vaccine serotypes (NVT). A small figure was inserted in the upper-right corner to show the proportion of PCVs covered serotypes and NVT in all isolates.

**Fig 2:**
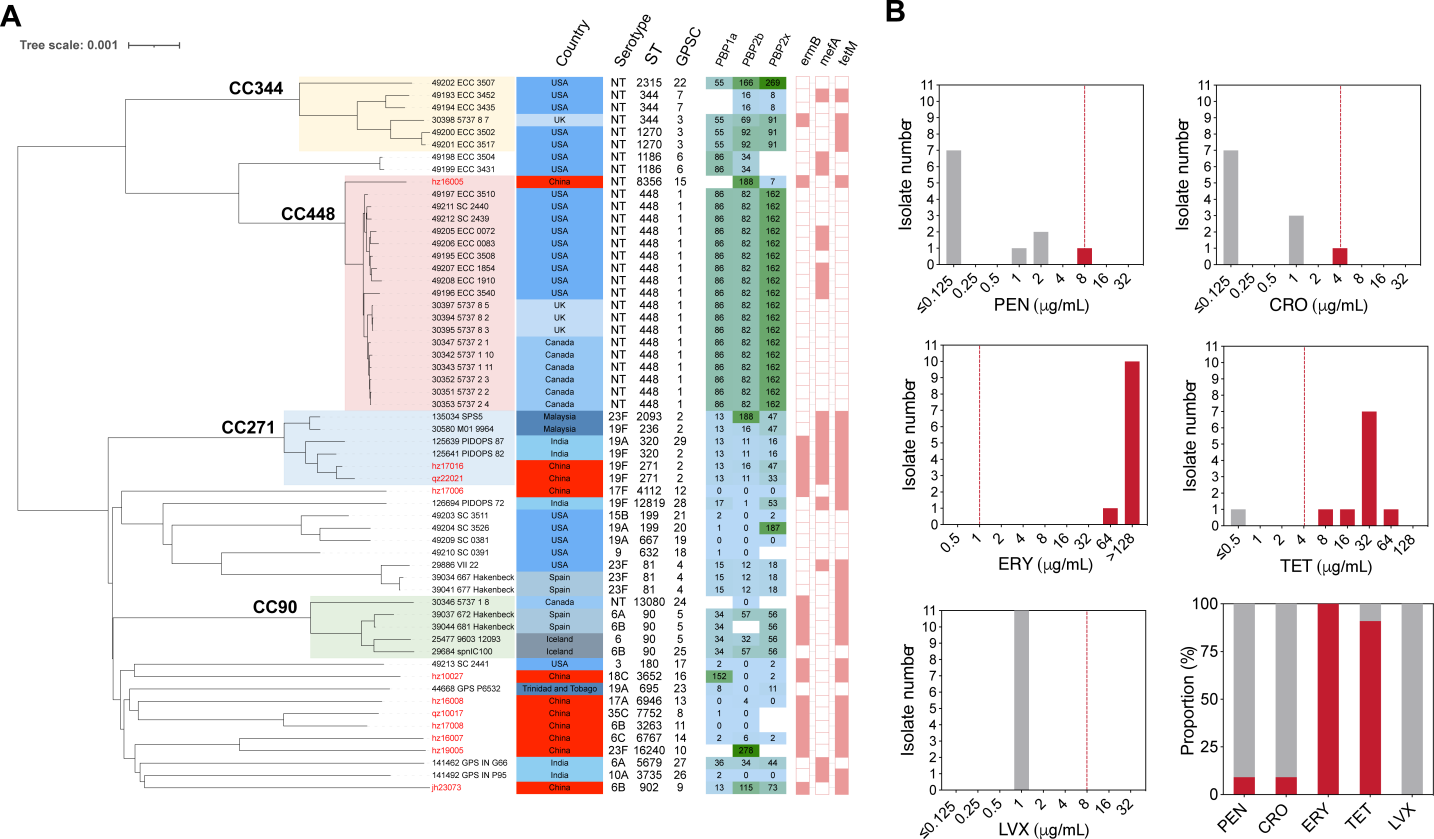
The phylogenetic analysis and distribution of major antimicrobial resistance determinants among global ocular *S. pneumoniae* strains and the antimicrobial resistance profile of pneumococcus in the current study. (**A**) The phylogenetic tree of global pneumococcal isolates from ocular infections (*n* = 58) was constructed via PopPUNK (v2.4.0) and aligned with the metadata, including country, serotype, ST type, GPSC type, and PBP1a-2b-2x mutation type, and antimicrobial resistance determinants (*ermB, mefA, and tetM*). This panel was visualized using iTOL (v6). (**B**) The antimicrobial susceptibility test results of PEN, CRO, ERY, and LVX against isolated ocular pneumococcus (one figure for each drug), and the last figure (bottom right corner of panel B) represents the resistance proportion in all tested pneumococcal strains against each drug. Red bars represent the resistance strains, and gray bars show the susceptible strains. The red dashed line in each figure indicates the breakpoint of each drug against *S. pneumoniae* according to the CLSI standard 2023.

Nine serotypes were identified in 11 isolates (Table 1). Serotypes 6B and 23F were determined in two strains each, and serotypes 6C, 17A, 17F, 18C, 19F, 35C, and NT were determined in one strain each. The serotype distribution data are presented in Fig. 1, where the 13-variant pneumococcal conjugate vaccine (PCV13) covered 54.5% of the detected serotypes and PCV20-added serotypes were not identified. Non-vaccine type (NVT) strains were found in four isolates, accounting for 45.5% of all pneumococcal isolates. Based on the non-meningitic breakpoint, one strain (qz22021) was resistant to PEN (MIC = 8 µg/mL) and CRO (MIC = 4 µg/mL). All strains resistant to ERY (MIC ≥ 64 µg/mL) and TET, except for one (qz10017), were susceptible to TET. All the isolated pneumococcal strains were susceptible to LVX (MIC = 1 µg/mL). The resistance rates of the pneumococcal strains against PEN, CRO, ERY, TET, and LVX were 9.1%, 9.1%, 100%, 91%, and 0%, respectively (Table 1; Fig. 2B).

### Phylogenetic analysis of worldwide pneumococcal isolates from ocular infections

We obtained genome sequence data for all *S. pneumoniae* isolates from eye swabs through the PubMLST database. A maximum likelihood phylogenetic tree was constructed, and metadata—including country, serotype, ST, Global Pneumococcal Sequencing Cluster (GPSC), PBP type, and genes encoding ERY and TET resistance (*ermB*, *mefA*, and *tetM*)—were aligned with each strain at the tips of the tree (Fig. 2A). We found four major clone complexes (CC) in all the analyzed strains: CC344, CC448, CC271, and CC90. The isolates from China did not fall within the four predominant clones, with only two strains originating from the major clone, CC271 (hz17016 and qz22021). Our strains predominantly exhibited sporadic distribution, with neither serotype nor STs concentrated. One NT strain was identified as the predominant clone, CC448, with an ST of 8356.

### Antimicrobial resistance and virulence gene detection

**Fig 3:**
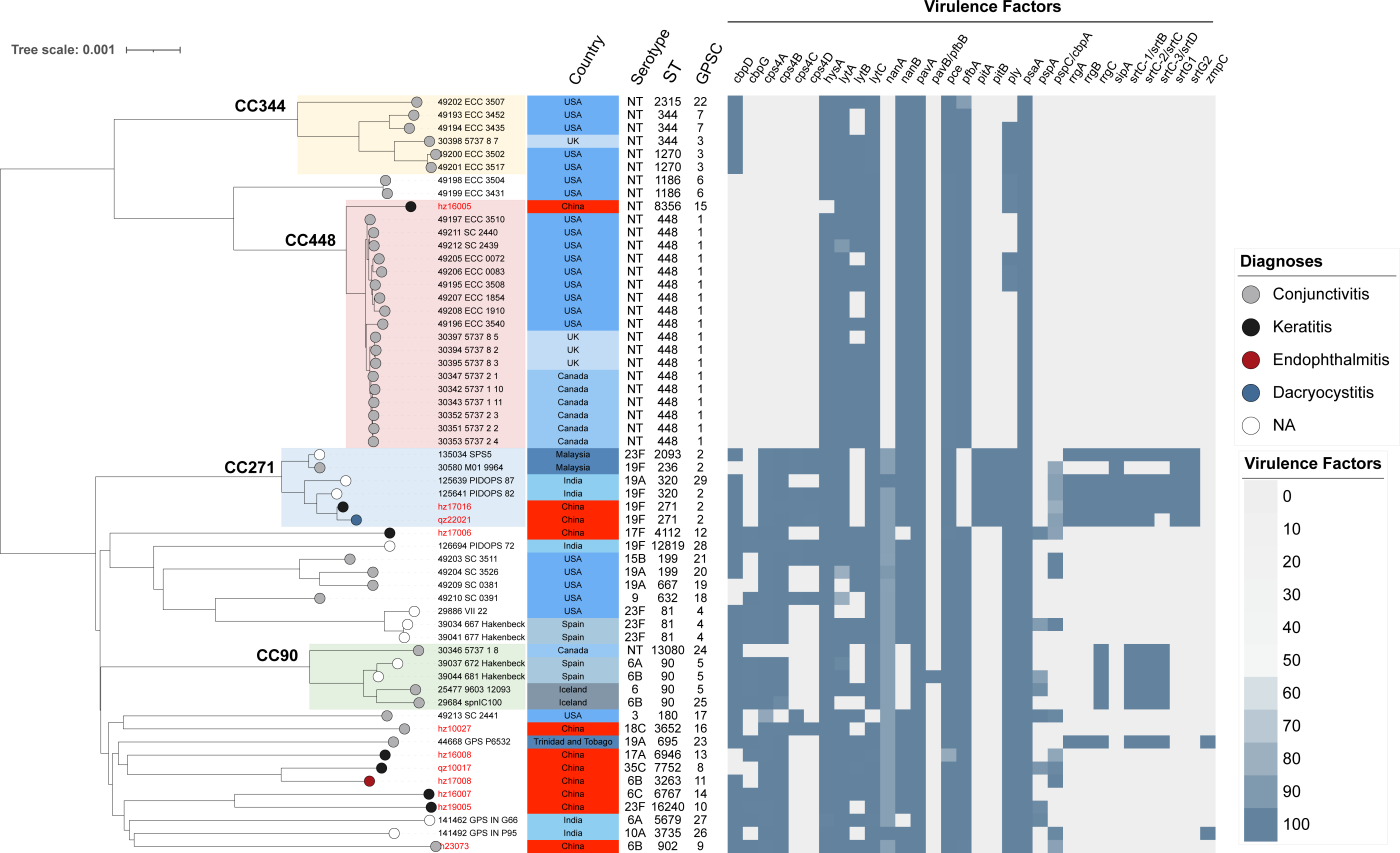
Clone distribution aligned with diagnoses and virulence factors detected in global ocular *S. pneumoniae*. The phylogenetic tree of global pneumococcal isolates from ocular infections (*n* = 58) was constructed via PopPUNK (v2.4.0) and aligned with the metadata, including diagnoses, country, serotype, ST type, GPSC type, and virulence factors. Four major clones were identified and shaded with different colors on the branch, including CC344 (light yellow), CC448 (light red), CC271 (light blue), and CC90 (light green).

As shown in Fig. 2A, PBP1a-2b-2x type 86-82-162 is the predominant type in all the analyzed strains. The PEN- and CRO-resistant strains (qz22021) belong to CC271 and carry PBP1a-2b-2x type 13-11-33, which differs from all other strains. ERY and TET resistance gene screening indicated that most isolates from China did not carry *mefA*, but all encoded *ermB* and *tetM*, which differs from pneumococcal isolates from other countries. For example, most strains from the United States and Canada do not carry *ermB* and encode *mefA* for ERY resistance. Moreover, the results of the virulence factor determination (Fig. 3) showed a clone specificity pattern for the four major clones. No specific virulence genes were detected in any ocular infection type. Most isolates from China did not carry *rrgABC*, *srtBCD*, or *zmpC*. This diversity suggests a complex evolutionary history and potential for adaptive variation in ocular infection pneumococcal isolates from different regions.

### Global recombination analysis of *S. pneumoniae* related to ocular infection

**Fig 4:**
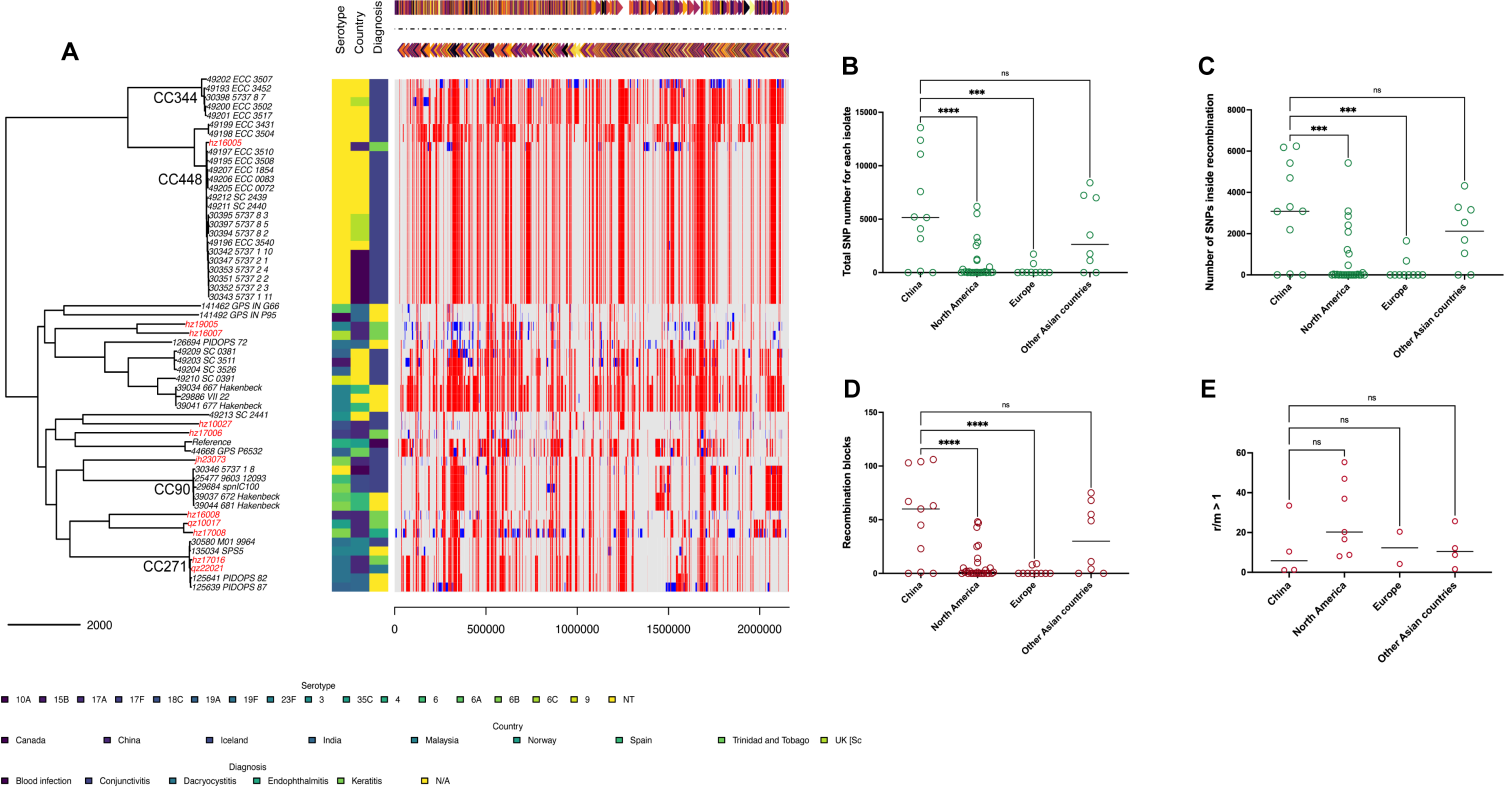
Recombination analysis of global ocular *S. pneumoniae.* (**A**) The phylogenetic tree of global pneumococcal isolates from ocular infections (*n* = 58) was constructed using the SNP of the core genome of each strain, and TIGR4 was used as the reference strain (aligned at the top of panel A). The ocular pneumococcal strains from China were marked in red in the isolated ID. The serotype, country, and diagnosis data were aligned at the tree tip. Recombination data were presented in red (recombination within each clone complex on an internal branch) and blue (recombination that occurred on a terminal branch that is unique to each isolate) blocks. Due to the high diversity of serotypes, countries, and diagnostic data presented in this figure, we chose to use a gradient color scale, ranging from bright yellow to dark blue, to illustrate the differences in the data. For example, in the serotype data, NT is bright yellow, serotype 3 is green, and 10A is dark blue. Specific data corresponding to each color are presented in the legend below panel A. The total SNP number (**B**), number of SNPs inside recombination (**C**), and recombination blocks (**D**) of each isolate were calculated in different regions (China, North America, Europe, and other Asian countries) worldwide. (**E**) The strains with r/m > 1 (the ratio of SNP introduced by recombination and by mutation) were also counted in the above four regions worldwide. “***” represents a *P* < 0.001, “****” represents a *P* < 0.0001, and “ns” indicates no significant difference between compared groups.

To further understand the distinct evolutionary pattern in China, we conducted recombination analysis of global ocular *S. pneumoniae* (Fig. 4A). Each red block represents a recombination region shared by the clone or branch, and the blue blocks represent recombination events detected at the tip of the branch that only occurred in one strain. Most pneumococcal strains from our collection contained recombination regions, which were obviously different from strains isolated in other regions worldwide. As shown in Fig. 4, the total number of ocular pneumococcal strains in China is significantly (*P* < 0.001) higher than those in North America and Europe (Fig. 4B). The same results were observed for the number of SNPs in the recombination region and recombination blocks (Fig. 4C and D). No difference was observed for r/m > 1 (ratio of recombination- and mutation-introduced SNP) (Fig. 4E).

### Global SNP analysis of *S. pneumoniae* related to ocular infection

**Fig 5:**
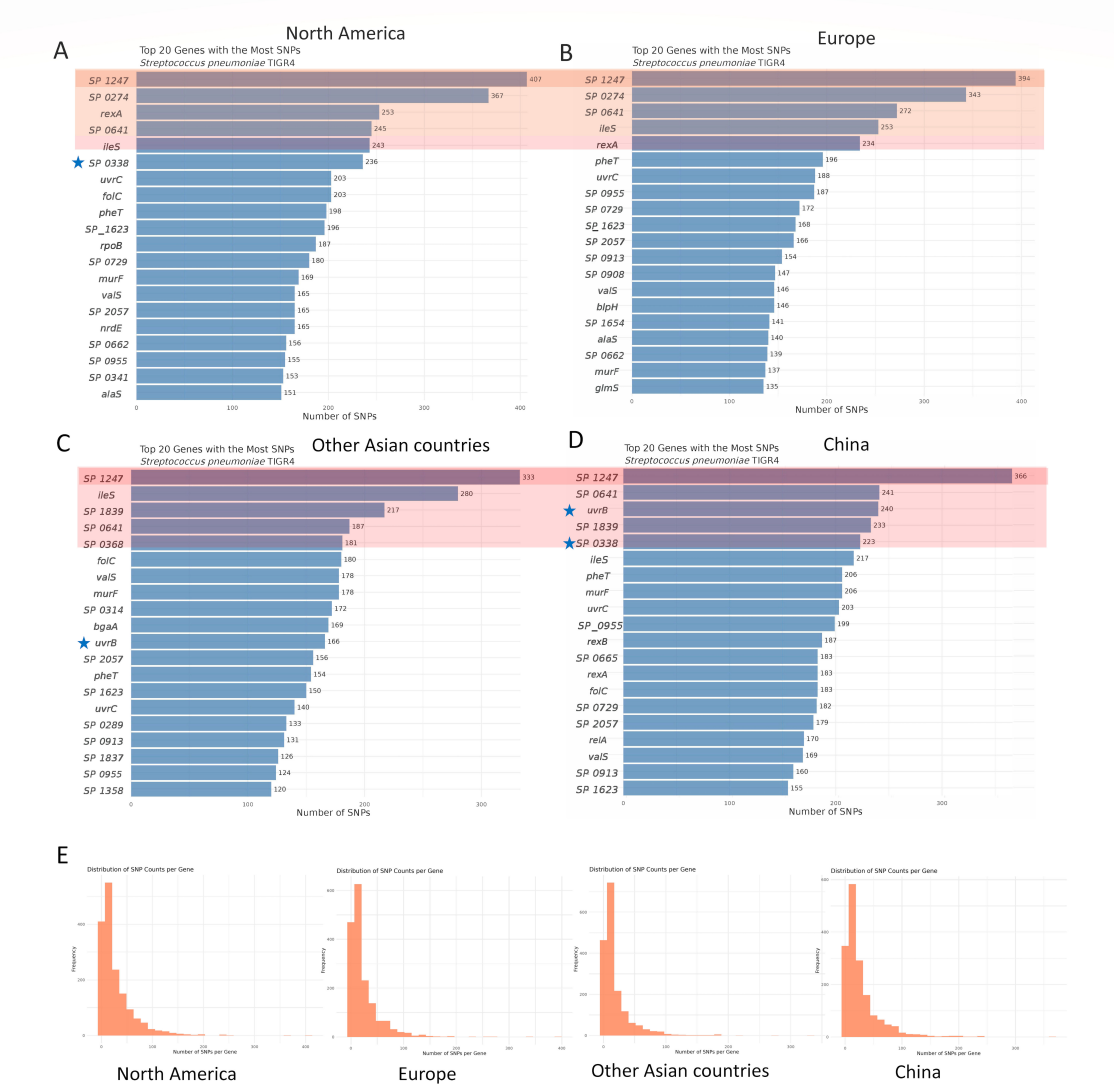
Single-nucleotide polymorphism analysis of global ocular *S. pneumoniae*. (**A–D**) The top 20 SNPs accumulated genes in ocular *S. pneumoniae* isolated from North America (**A**), Europe (**B**), other Asian countries (**C**), and China (**D**). The light red shading indicates the top five mutated genes. The dark red shading indicates the top mutated gene. The blue star marks gene ranked in the top five in China and not in other regions. (**E**) The SNP distribution across all genes of ocular *S. pneumoniae* from North America, Europe, other Asian countries, and China.

Globally, *SP_1247* is the most polymorphic locus, accumulating 333-407 SNPs in every ocular collection, regardless of ST or serotype (Fig. 5A through D). The top five most mutated genes were the same among the pneumococcal isolates from North America and Europe (Fig. 5AB). The isolates from China had the five most similar mutated genes to those from the rest of Asia. Within Asia, two genes (*uvrB* and *SP_0338*) were among the top five genes in China. The gene *uvrB* is the top six in North America, and *SP_0338* is the top 11 in other Asian countries. The top 20 most mutated genes in Europe did not contain these two genes. *SP_0665* and *relA* were only detected in ocular isolates from China. The distribution of SNPs in all genes is presented in Fig. 5E, where a similar result was observed, with the largest number of genes containing approximately 10 SNPs in all ocular pneumococcal isolates.

## DISCUSSION

PCV7/PCV13 injections significantly reduced pneumococcal conjunctivitis rates in children under 2 years old, with herd protection in infants under 6 months of age (32). Among the 11 ocular *S. pneumoniae* isolates, more than half belonged to PCV13, whereas PCV20-added serotypes were less prevalent in this population. Because of the small sample size, these results are purely descriptive and cannot be extrapolated to the broader population. Nevertheless, this pilot set still provides a serotype snapshot specifically for ocular pneumococcal disease in our region. The descriptive data also establish a feasible sampling and sequencing workflow that can be scaled to multicenter surveillance, where serotype-specific efficacy against sight-threatening infections can be formally assessed.

Regarding the drug resistance profile and mechanisms, only one PEN- and CRO-resistant strain was detected, which belongs to CC271 and has the PBP1a-2b-2x type 13-11-33, the major PBPs mutation combination type that causes β-lactam resistance and is widely disseminated in China (25, 33, 34). The difference in resistance gene profiles between Chinese isolates (carrying *ermB*) and those from the United States and Canada (carrying *mefA*) reflects a similar macrolide resistance profile difference in pneumococcus from all types of infection (35, 36). This could be due to variations in antibiotic usage and selective pressure. There is another report from China that studied ocular pneumococcus in children under 2 years old. They have reported that more than 70% of tested isolates were multi-resistant to tested antimicrobials (ERY, clindamycin, TET, and trimethoprim-sulfamethoxazole), which is consistent with our findings (37). Therefore, continuous monitoring of the evolution and international spread of these resistant clones, particularly CC271, is crucial for preventing pneumococcal ocular infections.

Non-encapsulated strains are the major pneumococci in conjunctivitis (4). Neuraminidase (NanAB) and zinc metalloproteinase (ZmpC) activities are increased in pneumococcal conjunctivitis (14, 38, 39). However, our data showed no NT strains for conjunctivitis, and two conjunctivitis pneumococcal isolates encoded serotypes 6B and 18C capsules, both carrying *nanAB,* while none encoded *zmpC*. A large-scale epidemiological study of pneumococcal conjunctivitis is needed to illustrate the different virulence factor-encoding patterns in Chinese conjunctivitis cases. Pneumolysin (PLY), NanAB, and ZmpC have been reported to be necessary for the initiation and development of keratitis (12, 38, 39). While we detected the *ply* gene in all keratitis pneumococcal isolates, one lacked NanB, and none encoded ZmpC. The region-specific pattern observed in the virulence factor-encoding pattern indicates that certain genetic lineages may have evolved specific traits that enhance their ability to cause disease.

Phylogenetically, we identified four major CC among the analyzed strains, suggesting that these clones are widespread and may have specific characteristics that have allowed them to become dominant in certain regions, such as CC344 and CC448 in the United States, the United Kingdom, and Canada; CC271 (dominant clone) in China, India, and Malaysia; and CC90 in Spain and Iceland. Interestingly, except in the United States, the United Kingdom, and Canada, none of the conjunctivitis isolates belonged to CC344 and CC488 NT strains. Previous observations of unencapsulated *S. pneumoniae* in conjunctivitis patients belonging to a unique cluster may only represent ocular pneumococcal epidemic characteristics in Western countries (4, 9). Moreover, the sporadic distribution of Chinese strains with no serotypes or clones implies a high level of genetic diversity. These clonal patterns are derived from a limited set of ocular isolates and should therefore be confirmed by larger regional surveys.

The unique recombination regions in our collection are markedly different from those isolated from other global regions. The significantly higher number of SNPs in ocular pneumococcal strains from China was corroborated by the elevated number of SNPs within recombination regions and recombination blocks in the Chinese strains. The frequency of recombination-induced evolution of ocular pneumococcus may be globally similar. However, the strains from China and other Asian countries are obviously on a different path than those from North American and European countries in terms of recombination sites and SNPs induced by each recombination event. It should be noted that these comparisons are based on 58 ocular isolates globally; the limited sample sizes cannot address population-level inferences. However, as no larger ocular-specific recombination data set exists, this first snapshot still provides a reference point for future multicenter studies. We would not refer to any other available ocular pneumococcal recombination analysis reports for comparison with our data. In our previous report, PCV availability in China led to fluctuating variations in vaccine-type respiratory pneumococcal genetic recombination (40). However, in the current study, the vaccine-covered serotypes only accounted for half of our isolates, and most of the strains from North American and European countries were NT strains. PCV stress may not be the pressure for recombination differences. We believe that these differences can be attributed to geographical or host-specific influences.

*In silico* analysis showed that the gene *SP_1247*, which accumulates the highest number of SNPs in every ocular collection worldwide, encodes the chromosome segregation protein SMC, which condenses and segregates the bacterial chromosome during cell division (41). This pattern raises the possibility that cell proliferation and DNA segregation kinetics are under positive selection within the ocular niche, where oxidative stress and frequent light exposure continuously modify the pneumococcal genome to adapt to the environment. In contrast to other regions, *uvrB* and *SP_0338* were the top five mutated genes exclusively in the Chinese isolates. The gene *uvrB* in *S. pneumoniae* has been reported to be highly conserved compared with that in *E. coli*, where the UvrAB complex is used for the search and recognition of UV-damaged DNA (42). *SP_0338* encodes the ATP-binding subunit of a putative Clp protease that degrades misfolded and oxidatively damaged proteins. Nucleotide excision repair is coupled to ATP-dependent degradation of oxidized or misfolded proteins by the Clp protease (43). Collectively, the convergent targeting of *SP_1247* (DNA integrity), *uvrB* (DNA damage repair), and *SP_0338* (protein homeostasis) defines a candidate genomic signature whose adaptive value remains to be tested. To our knowledge, this is the first description of these three loci as mutational hotspots in ocular pneumococci. Their biological relevance can only be established through targeted mutagenesis and functional assays.

This study was a retrospective analysis of 11 ocular *S. pneumoniae* isolates, and the sample size may have limited the power of our findings. However, the isolates collected in this study were scattered across different lineages in the context of global ocular genome analysis and all exhibited the same pattern of recombination blocks occurring recently (at the tip of the tree branch). This comparison reveals that these isolates are not the result of a single local outbreak but rather represent sporadic introductions of globally circulating strains into our hospital. Although the absolute number of isolates was small, their genetic heterogeneity and identical tip-located recombination signatures indicated that they captured the current global pneumococcal distribution, thereby mitigating the concern that our findings were limited to a small sample size.

Our research provides a comprehensive global genomic investigation into the molecular epidemiology of ocular pneumococcal infections. This revealed a distinctive evolutionary pattern in Asian countries, differentiating them from ocular strains isolated from other global regions. Moreover, distinct recombination regions in Asian strains can harbor genes that confer specific adaptive advantages to the local population, such as enhanced host immune evasion or tissue tropism. Notably, our genomic survey identifies DNA integrity maintenance, UV-damage repair, and protein homeostasis as mutational hotspots in ocular pneumococci. Whether these pathways constitute virulence determinants remains to be established through experimental validation. Comprehensive investigations in a global context are warranted to elucidate their underlying dynamics and pathogenicity.

## Data Availability

All sequence data of the whole-genome sequenced ocular pneumococcal strains collected in the current study were uploaded to the NCBI under the BioProject no. PRJNA1119743.
